# Production of Alkaline Protease by Solvent-Tolerant Alkaliphilic *Bacillus circulans* MTCC 7942 Isolated from Hydrocarbon Contaminated Habitat: Process Parameters Optimization

**DOI:** 10.1155/2013/942590

**Published:** 2013-11-28

**Authors:** Ulhas Patil, Ambalal Chaudhari

**Affiliations:** ^1^Department of Microbiology, R. C. Patel Arts, Commerce and Science College, Shirpur 425 405, India; ^2^School of Life Sciences, North Maharashtra University, Jalgaon 425 001, India

## Abstract

In the present investigation, a newly isolated organic solvent-tolerant and alkaliphilic bacterial strain was reported from a hydrocarbon (gasoline and diesel) contaminated soil collected from the petrol station, Shirpur (India). The strain was identified as *Bacillus circulans* MTCC 7942, based on phenotype, biochemical, and phylogenetic analysis of 16S rRNA gene sequence. The capability of *Bacillus circulans* to secrete an extracellular, thermostable, alkaline protease and grow in the presence of organic solvents was explored. *Bacillus circulans* produced maximum alkaline protease (412 U/mL) in optimized medium (g/L): soybean meal, 15; starch, 10; KH_2_PO_4_, 1; MgSO_4_·7H_2_O, 0.05; CaCl_2_, 1; Na_2_CO_3_, 8; pH 10.0 at 37°C and 100 rpm. The competence of strain to grow in various organic solvents—*n*-octane, dodecane, *n*-decane, N,N-dimethylformamide, *n*-hexane, and dimethyl sulfoxide, establishes its potential as solvent-stable protease source for the possible applications in nonaqueous reactions and fine chemical synthesis.

## 1. Introduction

The world enzyme market will grow up to $2.1 billion by 2016 [1]. Of these, alkaline protease alone accounts for 40% of the total world enzyme production, with growing applications in bakery, brewing, detergent, diagnostic reagents, feeds modification, leather finishing, laundry additives, pharmaceuticals, peptide synthesis, silk, silver recovery from X-ray/photographic film, soy processing, and waste treatment [2].

Majority of alkaline proteases produced by mesophilic microbes are effective in a narrow range of pH, temperature, and ionic strength, accordingly unsuitable for commercial purpose under demanding industrial conditions. Hence, alkaline proteases secreted by alkaliphilic bacteria are of potential interest in detergent industry due to their ability to withstand extremes of temperature (40–60°C), alkaline pH (9–11), high salt concentration, and other harsh conditions [3].

Alkaline protease that catalyses peptide bond formation in the nonaqueous media has greatly expanded potentials in green chemistry for fine chemical synthesis [4]. Alkaline proteases have expanded new possibilities such as (i) shifting of thermodynamic reaction equilibrium to favour synthesis, (ii) enhancement of bioavailability of hydrophobic substrates, (iii) total inhibition of water-dependent side reactions, (iv) alteration in enantioselectivity of reaction, (v) improving thermal stability, and (vi) facilitating the product recovery. Thus, the stability of proteases in organic solvents offers certain merits like (i) synthesis of various compounds, (ii) bioremediation of waste containing organic solvents, and (iii) possibility of newer reactions like transesterification, ammonolysis, and thiolysis. Hence, the search for alkaline proteases stable to harsh conditions is of great significance. This constitutes the main objective of the present study.

Microbial diversity of exotic environment including man-made biotopes offers an unconditional source to screen versatile microbes with inherent stable proteases. The naturally robust protease capable of functioning in harsh conditions is anytime suitable than artificially stabilized enzyme for a biocatalysis in the industry.

Microbe develops an array of physiological and molecular adaptations for survival in virtually every extreme environment. Although, the stability of alkaline protease in the presence of organic solvents has been improved by approaches like physical, chemical modification, immobilization, protein engineering, and recombinant DNA, the selection of microbes secreting alkaline proteases naturally endowed with stability and activity in nonaqueous media from natural or man-made extreme environment can serve the purpose of industry [5, 6]. In this regard, several solvent-tolerant alkaline protease producers, mainly *Bacillus *sp. and *Pseudomonas *sp., from extreme natural habitats have been reported [7–11].

In view of the above facts, the present work describes isolation of a new organic solvent-temperature-tolerant strain of *Bacillus circulans* from man-made contaminated habitat with emphasis on the optimization of physical and nutritional requirements for the effective production of organic solvent-stable and extracellular alkaline protease.

## 2. Materials and Methods

### 2.1. Microorganism

The newly isolated strain (UB2) was identified on the basis of a battery of morphological and biochemical characteristics. The preliminary identification was confirmed by (i) Microbial Type Culture Collection (MTCC), Chandigarh using Biolog microbial identification system (Biolog automated Microstation system, USA), and (ii) taxonomic characterization using nucleotide sequence of 16S rRNA gene as per Sambrook et al. [12]. The strain was maintained on nutrient agar slants at 4°C.

### 2.2. Effect of Organic Solvents on Isolated Strain

The organic solvent tolerance of *Bacillus circulans* MTCC 7942 was analysed as per Ogino et al. [13]. The organic solvent tolerance against chloroform, acetone, butanol, xylene, benzene, toluene, *n*-octane, dodecane, *n*-decane, N,N-dimethylformamide, cyclohexane *n*-hexane, and dimethyl sulfoxide was confirmed by procedure as described previously [10]. The strain was also cultivated in the absence of organic solvent in an Erlenmeyer flask with a rubber stopper as positive control.

### 2.3. Protease Assay

Alkaline protease activity, using buffered casein as a substrate was assayed as per Nakanishi et al. [14] with several modifications as described earlier [10]. One unit of alkaline protease activity (U) was defined as that amount of enzyme required to produce peptides equivalent to 1.0 *μ*g of tyrosine in the filtrate per minute per mL at pH 10.0 and 60°C.

### 2.4. Protein Determination

The culture free broth was precipitated with 6 N trichloroacetic acid. The suitably diluted protein aliquot was used for determination of protein concentration at 750 nm as per Lowry et al. [15] using bovine serum albumin (BSA) as a standard.

### 2.5. Optimization of Production Medium Ingredients for Protease Production

Various carbon sources (glucose, fructose, xylose, sucrose, starch, maltose, and molasses) were analysed for highest protease production. Also, various nitrogen sources (beef extract, bovine serum albumin, casamino acids, casein, feather meal, gelatin, peptone, soybean meal, and yeast extract) were scrutinized for enhanced alkaline protease production. For this, each nitrogen ingredient (1%) was separately added to the basal medium containing (g/L): starch, 20; KH_2_PO_4_, 1; MgSO_4_·7H_2_O, 0.05; CaCl_2_, 1; Na_2_CO_3_, 8. The effect of medium supplementations with different concentrations of glucose (0–2% w/v), starch (0–2% w/v), soybean meal (0–2% w/v), sodium carbonate (0-1% w/v), KH_2_PO_4_ (0–0.2% w/v), and CaCl_2_ (0–0.2% w/v) were evaluated under similar conditions.

### 2.6. Optimization of Physical Parameters for Protease Production

The effect of pH on growth and protease production was examined by growing the strain in a medium containing (g/L): soybean meal, 10; starch, 20; KH_2_PO_4_, 1; MgSO_4_·7H_2_O, 0.05; CaCl_2_, 1; at 37°C and 100 rpm at varying pH (7.0, 8.0, 9.0, 10.0, and 12.0). A separately sterilized sodium carbonate (0.2–1.0% w/v) was used to adjust alkaline pH of the medium. The influence of temperature (30–60°C) was analysed for growth and protease secretion in the same medium discussed above, at pH 10.0, for 48 h. The effect of agitation on growth and protease production was also examined by growing the strain in medium at pH 10.0, 37°C for 48 h under various speeds of rotation (0–200 rpm). For preparation of inoculum, a prepared suspension (0.5 absorbance at 660 nm) was evaluated in the range of 2–10% (v/v).

### 2.7. Growth and Protease Production Profile

The growth and protease production were examined in the optimized production medium containing (g/L): soybean meal, 15; starch, 10; KH_2_PO_4_, 1; MgSO_4_·7H_2_O, 0.05; CaCl_2_, 1; Na_2_CO_3_, 8; pH 10.0 at 37°C and 100 rpm by analyzing samples at 6 h intervals. In each experiment, cell density was determined by harvesting biomass after centrifugation (12,000 rpm, 4°C and 10 min) and washing twice with cold distilled water. The washed biomass was dried under vacuum at room temperature until a constant weight was attained.

### 2.8. Statistical Analysis

Each experiment was carried out in triplicate set; results were presented as mean ± SD. The results were considered as statistically significant when *P* < 0.05. The appropriate vertical error bars are displayed on each graph. The error bar values were based on standard deviations, calculated for each data point by using SigmaStat software and Microsoft Excel.

## 3. Results and Discussion

### 3.1. Identification of Strain

The potential organic solvent tolerant bacterial isolate UB2 was isolated from soil collected from petrol station (nearly 25 years old) located at Shirpur (Dist-Dhule), India [21° 21′ 0′′ N/74° 53′ 0′′ E]. The sample soil was found moistened with gasoline and diesel due to spills and emits typical petroleum odour. Petrol consists mostly of aliphatic hydrocarbons. It also contains the aromatic hydrocarbons like toluene and benzene to increase its octane rating. Microorganisms present in petroleum contaminated soils and their role in the petroleum product degradation have been reported earlier [16, 17]. The present investigation has revealed the presence of solvent-tolerant, alkaliphilic bacteria in man-made, petroleum compound contaminated soil.

**Figure 1:**
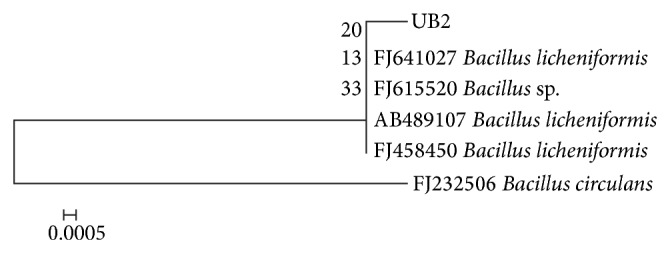
Phylogenetic tree of strain UB2 showing relatedness with* Bacillus *sp.

Morphological, cultural, and biochemical characteristics of UB2 strain are summarized in Table 1. On the basis of these characteristics, the newly isolated UB2 strain was identified as *Bacillus circulans. *This identification was further verified by the Biolog system at Microbial Type Culture Collection (MTCC), Chandigarh. The assigned MTCC accession number is 7942. The 16S rRNA gene of strain UB2 was PCR amplified, purified PCR product sequenced and aligned by using BLAST analysis on the NCBI server (http://www.ncbi.nlm.nih.gov/BLAST). To confirm the identity of UB2, 888 bp 16S rRNA genes were sequenced, respectively, and submitted to the NCBI Gene bank (accession number UB2: GQ461354). For comparison, currently available sequences at NCBI were used and multiple sequence alignment performed by using Bioedit 7.0. The phylogenetic tree was generated; the result showed that UB2 is related to *Bacillus *spp. (Figure 1).

### 3.2. Effect of Various Organic Solvents on Isolated Strain

**Figure 2:**
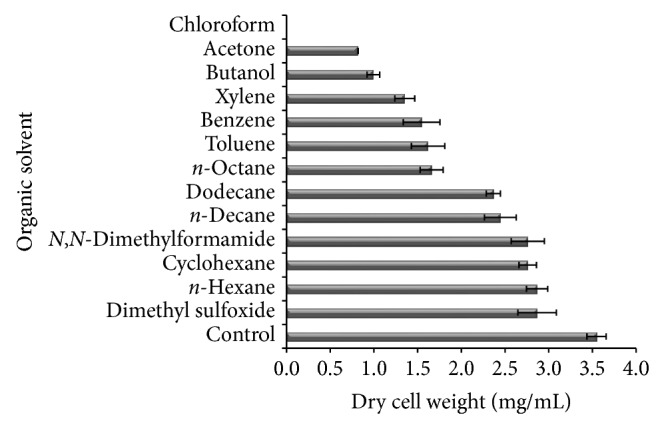
Dry cell weight of strain UB2 cultured in 100 mL of the nutrient liquid medium and 30 mL of organic solvent for 72 h.

The effect of various organic solvents on the growth of *Bacillus circulans* was investigated by measuring the dry weight of the culture.  Figure 2 showed the dry cell weight of strain *B. circulans* after 72 h cultivation in the presence of different organic solvents. The strain was able to grow in all solvents studied except chloroform. Organic solvents are usually toxic to microorganisms, but several bacteria tolerant to organic solvents and able to produce protease have been reported [11, 12, 18–21]. Generally, organic solvents are toxic to living organisms, because of their adverse effects on biological membranes while several researchers correlated solvent toxicity with the hydrophobic character of the solvent, expressed by the logarithm of its partition coefficient between octanol and water, denoted by a log *P* value [12, 22]. Generally, solvents with log *P* values below 4 are considered extremely toxic as their degree of partitioning into the aqueous layer is higher [23]. Therefore, the presence of solvents may lead to a reduction in the growth. However, cells which are adapted to solvents can achieve the maximum growth rate in the presence of solvents.

Compared to the optimum growth in control, growth of *B. circulans* MTCC 7942 was (i) maximum in the presence of dodecane (log*P* = 6.6), *n*-decane (log*P* = 5.6), *n*-hexane (log*P* = 3.6), cyclohexane (log*P* = 3.2), DMF (log*P* = −1.0), and DMSO (log*P* = −1.4), (ii) moderate in the presence of octane (log*P* = 4.5), xylene (log*P* = 3.1), benzene (log*P* = 2.0), and toluene (log*P* = 2.5), (iii) less in the presence of *n*-butanol (log*P* = 0.8) and acetone (log*P* = −0.2). No growth was detected in the presence of chloroform (log*P* = 2.0). The number of bacteria showing organic solvent tolerance is limited; the previously studied organic solvent-tolerant strains were *Bacillus *sp. [24], *B. pumilus *[8], *B. licheniformis *[25], and *B. sphaericus *[26]. Chloroform strongly inhibits the growth of *B. circulans* studied in present investigation; this might be due to a reduction in the affinity of the cells for nutrient and change in the structure of cell envelope [22]. Since the water-miscible solvents (DMF and DMSO) are usually used in organic reactions, *B. circulans* might have potential applications in nonaqueous reactions.

### 3.3. Effect of Various Media Ingredient on Growth and Protease Production

**Figure 3:**
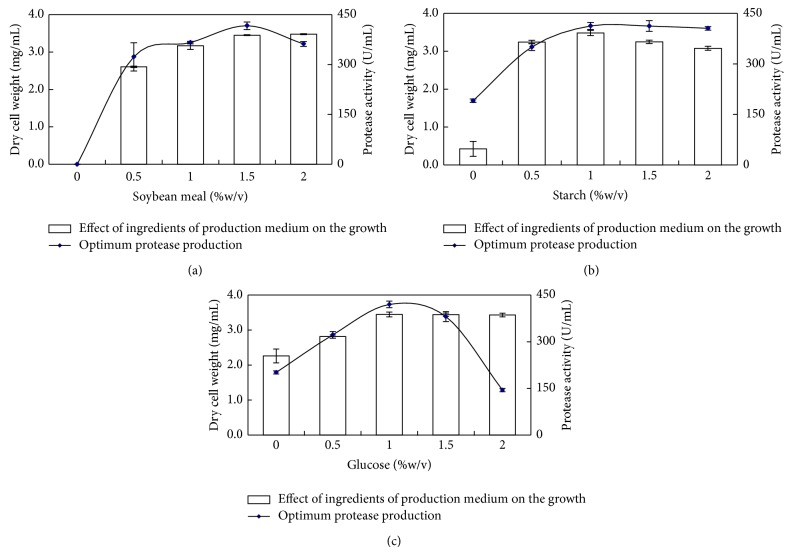
Effect of ingredients of production medium on the growth and optimum protease production by *B. circulans *MTCC 7942 (a) soybean meal; (b) starch; and (c) glucose.

Nitrogen is an essential component and has a profound effect on protease synthesis. Among various nitrogen sources, soybean meal exhibited noticeably higher protease production by *B. circulans *MTCC 7942 grown in basal medium containing (g/L) starch 20, KH_2_PO_4_, 1; MgSO_4_·7H_2_O, 0.05; CaCl_2_, 1, pH 10 at 37°C for 48 h, followed in decreasing order by peptone, gelatin, yeast extract, casamino acids, casein, and beef extract. Poor production of protease was observed with bovine serum albumin and feather meal (Table 2). Although the nitrogen sources used in this study contain varied concentration of total nitrogen (feather meal, 12% w/w; gelatin, 18% w/w; casein, 16% w/w; and soybean meal, 8% w/w), the soybean meal supported maximum protease yield. The complexity, solubility, and amino acid compositions are the factors probably affecting efficient utilization of these sources by the *B. circulans*. The free amino acids and peptides in digested nitrogen sources (peptone, yeast extract, casamino acids, and beef extract) could be responsible for the feedback repression of proteolytic system of the *B. circulans*. Hence, the most effective inducer of alkaline protease synthesis was soybean meal. Protease production by *B. circulans *MTCC 7942 attained maximum growth and maximum protease production at 1.5% soybean meal (Figure 3(a)).

Soybean meal being a comparatively cheap and readily available substrate, its industrial suitability for cost-effective production of protease at commercial level was a positive gain. Soybean meal was also reported to be a suitable nitrogen source for protease production [27], while a combination of casein (1%) with soybean meal (1.5%) gave the maximum protease production by *Bacillus horikoshii* [28]. On the contrary, the presence of yeast extract as a nitrogen source in the production medium had shown substantially enhanced protease production by *Bacillus acidophilus *subsp.* halodurans *[29]. Fujiwara et al. [30] recorded maximum enzyme production using a combination of 3.0% (w/v) soybean meal and 1.5% (w/v) bonito extract. Higher levels of protease production were reported from *Bacillus pseudofirmus* AL-89 and *Nesterenkonia* sp. AL-20, on feather meal [31].

Organic nitrogen medium alone was not sufficient for the production of protease; additional carbon source (glucose or starch) in the basal medium was essential for the optimum protease production of this strain. Protease production by *B. circulans *MTCC 7942 by employing various carbon sources is summarized in Table 3. The highest protease production of it was a function of glucose, maltose, and starch, followed by fructose and xylose. Further, optimum concentration of starch was evaluated by incorporating 0.5–2% (w/v) in basal medium containing (g/L) soybean meal 10, KH_2_PO_4_, 1; MgSO_4_·7H_2_O, 0.05; CaCl_2_, 1. The optimum concentration of starch for protease production was 1.0% (Figure 3(b)). Also, 0.5–2% glucose was analysed to find out the repression effect of it. The protease production of *B. circulans *MTCC 7942 was suppressed by glucose beyond 1.5% concentration (Figure 3(c)). Glucose and lactose were reported as effective carbon sources for protease production in *Bacillus *sp. AR-009 [32] and *B. licheniformis* ATCC 21415 [33]. Similarly, increased level of protease production by *B. pseudofirmus* AL-89 was observed upon addition of glucose [31].

**Figure 4:**
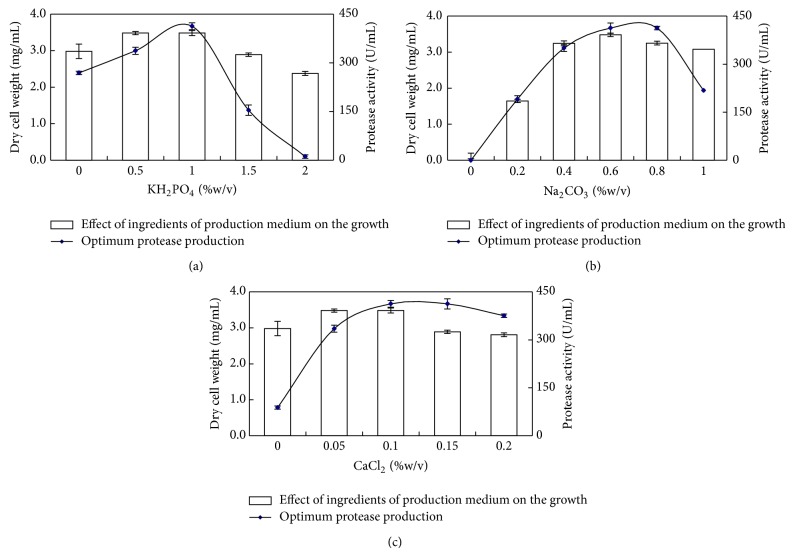
Effect of ingredients of production medium on the growth and optimum protease production by *B. circulans *MTCC 7942 (a) KH_2_PO_4_, (b) Na_2_CO_3_, and (c) CaCl_2_.

The effect of phosphate ions was examined for enhanced growth and production of protease by *B. circulans *MTCC 7942 (Figure 4(a)). In most studies, potassium phosphate has been used as a source of phosphate and buffering component of the medium [30, 34–36]. Phosphate at the concentration of 2 g/L was found optimal for protease production by *Bacillus firmus*, while further supplementation showed an inhibition of cell growth and repression in protease production. Also, phosphate concentration above 4 g/L leads to precipitation of the medium on autoclaving [34]. On the contrary, Phadatare et al. [37] showed that salts did not have any effect on alkaline protease production from *Conidiobolus coronatus*.

The effect of sodium carbonate on the growth and protease production by *B. circulans *MTCC 7942 was examined (Figure 4(b)). Sodium carbonate was detected as essential media component as in the absence of its growth does not occur. The optimum concentration which supported maximum growth and protease secretion was 0.6% (w/v). The role of Na_2_CO_3_ was to maintain an alkaline pH of the medium and ensure protease production was detected in *B. circulans*. An optimal protease production was observed at a concentration of 0.8% (w/v) sodium carbonate [38]. Horikoshi and Akiba [39] used sodium carbonate to adjust pH of the medium for alkaline protease production, while Genckal and Tari [40] used 6 M NaOH to set the alkaline pH of Horikoshi media for alkaline protease production using *Bacillus* L18 and L21. During production of protease, sodium carbonate incorporation as a source of alkalinity to their growth media is routinely practised so as to simulate the natural ecological habitat and enhance their growth. Calcium chloride was found to be a crucial component of production media for alkaline protease production by *B. circulans *MTCC 7942 (Figure 4(c)). CaCl_2_ has a vital effect in protease production by *B. circulans. *Similarly, CaCl_2_ accelerated the production of protease in *Streptomyces *[41],* Bacillus *sp. PCSIR EA-3 [42].

### 3.4. Effect of Physical Parameters on Growth and Enzyme Production

**Figure 5:**
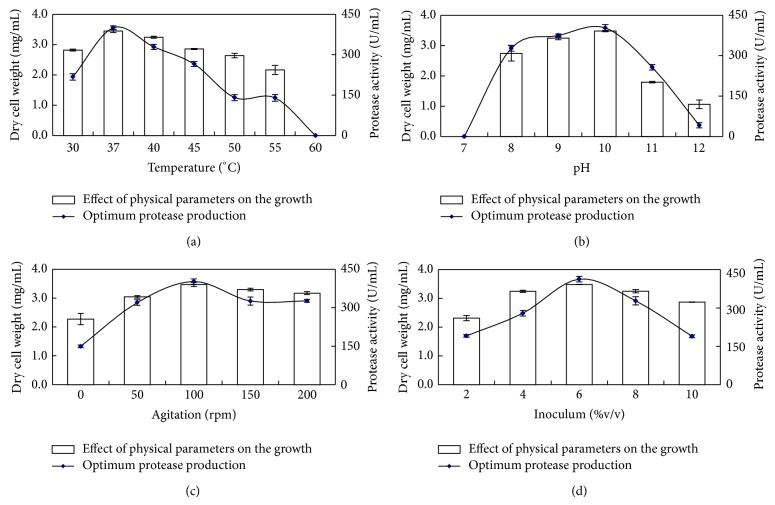
Effect of physical parameters on the growth and optimum protease production by *B. circulans *MTCC 7942 (a) temperature; (b) pH; (c) agitation; and (d) inoculum.

The effect of incubation temperature on growth and protease production by *B. circulans *MTCC 7942 was examined. Although strain could grow in broad temperature range 30–55°C, maximum growth and protease production was detected at 37°C suggesting thermotolerant nature of strain. The strain secreted alkaline protease even at 50–55°C, but its optimum secretion (401.08 U/mL) was at 37°C (Figure 5(a)). Several earlier reports have indicated incubation temperature as a critical parameter for regulating the synthesis and secretion of alkaline protease by microbes [43, 44], by affecting (i) energy metabolism and oxygen uptake [45], (ii) translational synthesis of protein [46], and (iii) the physical properties of cell membrane [20]. *Bacillus* sp. secreted proteases over a wide range of temperature (30–60°C) [2, 28, 39]. A critical assessment with the previous reports on the characteristics of alkaliphilic *Bacillus *strains producing alkaline proteases, revealed that several alkaliphilic *Bacillus *strains have temperature optima between 30 and 37°C and are mostly of mesophilic type [33, 47–49].

The pH of the medium has an effect on alkaline protease production. *B. circulans *MTCC 7942 grew maximally in the pH range of 8.0 to 11.0; no growth was detected at pH 7.0, indicating that the strain was an obligate alkaliphile. Although at pH 11.0 strain produced an appreciable protease yield, the optimum pH value for growth and alkaline protease production was pH 10.0 It secretes 403.2 ± 12 U/mL protease at pH 10.0 (Figure 5(b)). Interactions between the metabolic reactions and genetic regulatory mechanism(s), product and byproduct formation in the bioprocess for alkaline protease production are dependent on pH. The pH also influences the transport of various nutrient components across the cell membranes, which in turn sustain the cell growth and the product formation. Similar results were obtained by Rahman et al. [50] who reported maximum protease production in *B. stearothermophilus* at pH 10 and 60°C. Alkaline protease production was reported using *B. circulans *under solid state fermentations at pH 10.0 [51, 52], *Bacillus* sp. B001 [47] at pH 10 and *B. licheniformis* MP1 at pH 8 [48].

Effect of agitation on *B. circulans *MTCC 7942 was analyzed at 0, 50, 100, 150, and 200 rpm for 48 h. An increase in agitation speed (rpm) enhanced the growth of *B. circulans *MTCC 7942 with an increase in protease production up to 100 rpm. However, further increase in agitation did not contribute to the growth and protease production. Hence, the optimum agitation speed for growth and protease production was 100 rpm (Figure 5(c)). Agitation enhanced the protease production in this case, probably due to (i) better nutrient transfer rate, (ii) an increase in the solubility of oxygen through the mixing of the contents, (iii) dispersion of cell aggregates, and (iv) enhanced aerobic metabolism during fermentation [40, 49]. On the contrary, Frankena et al. [45] reported an increase in protease production in *B. licheniformis* when oxygen was limited. Production of alkaline protease was enhanced in (i) *Bacillus* sp. when agitated at 150 rpm [53] and (ii) *B. licheniformis* ATCC 21415 when the agitation rate increased from 250 to 400 rpm [33], while low or no agitation caused a drastic reduction in protease production in *B. firmus* [34] and *Bacillus mojavensis* A21 [54]. Compared to previously reported *Bacillus* strains, the studied strain *B. circulans *MTCC 7942 did not require higher speed (more than 100 rpm) for protease production, probably due to little requirement for oxygen.

### 3.5. Inoculum Optimization

It is essential to establish an effective inoculum development program, regardless of the scale of fermentation. Such program not only aids consistency on a small scale but is invaluable in scaling up the fermentation for providing homogenous, contamination free inoculum. The inoculum size had an impact on cell growth, minimization of contamination, and enzyme synthesis, depending on the characteristics of the strains. For preparation of inoculum, a suspension was prepared by mixing pregrown *B. circulans *MTCC 7942 with saline to achieve 0.5 absorbance at 660 nm. The effect of inoculum size was studied in the range 2–10% (v/v). Growth and protease production of *B. circulans *MTCC 7942 was maximum at 6% (v/v) inoculum incubated for 48 h (Figure 5(d)). An inoculation of 2–5% culture was an optimum value for *the Bacillus* type of strains reported in the literature, which supported our findings. Higher inoculum sizes might be responsible for lack of oxygen and early nutrient depletion. These include 1.0% (v/v) for *Bacillus* sp. [55], 5.0% (v/v) for *B. licheniformis* ATCC 21415 [33], and 10.0% (v/v) for *Bacillus* sp. [56].

## 4. Conclusions

**Figure 6:**
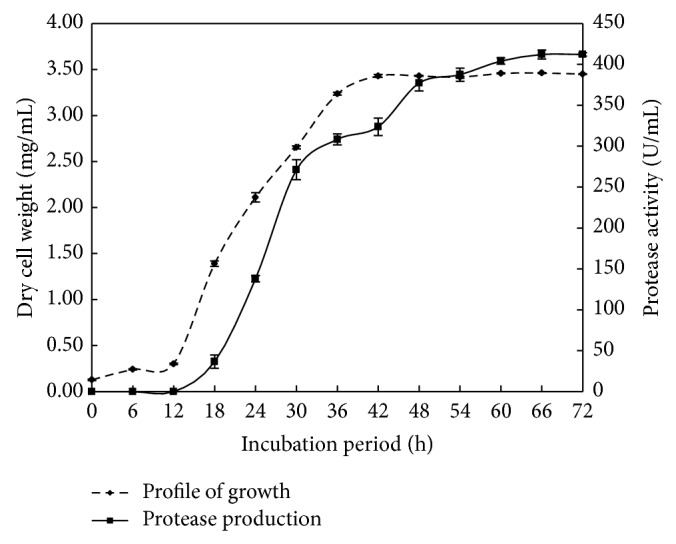
Profile of growth and protease production of *B. circulans *MTCC 7942 as a function of time.

Production of alkaline protease from solvent-tolerant, alkaliphilic* B. circulans *MTCC 7942, isolated from hydrocarbon contaminated soil, was reported. A time course study was performed to determine bacterial growth and protease production of *B. circulans *MTCC 7942.  Figure 6 shows that (i) logarithmic growth phase commenced at 12 h and continued thereafter until 36 h, (ii) maximum growth in biomass was attained until 48 h, (iii) protease production initiated just before 24 h continued until 60 h, attains maximum protease activity (412 U/mL), and (iv) remained steady in the medium even after 72 h, indicating its resistance to inactivation and stability of protease. This is a commercially desirable trait for maximum recovery during processing.

## Figures and Tables

**Table 1:** Morphological, cultural, and biochemical characteristics of UB2 strain.

Characteristic	Description
(A) Morphological	
Shape	Rod
Arrangement	Single
Gram stain	Gram positive
Motility	+
Endospore	+
Capsule	−

(B) Cultural	
Growth temperature (°C)	
15	−
25	+
37	+
40	+
42	+
45	+
55	+
65	−
Growth pH	
5.0	−
6.0	−
7.0	−
8.0	+
9.0	+
10.0	++
11.0	+
NaCl (% tolerance)	
2.5	+
5.0	−
7.0	−
8.5	−
10.5	−

(C) Biochemical	
Utilization of	
Malonate	+
Ribose	+
Xylose	+
L-Arabinose	−
Glucosamine	+
Inositol	−
Glucose	+
Fructose	+
Mannitol	+
D-Sorbitol	+
Maltose	+
Trehalose	+
Melibiose	−
Sucrose	+
Cellobiose	+
Lactose	−
Melezitose	−
Raffinose	−
Inulin	−
Enzyme	
Amylase	+
Arginine dihydrolase	+
Catalase	+
Gelatinase	+
Lysine dehydrogenase	−
Nitrate reductase	−
Ornithine decarboxylase	−
Oxidase	+
Urease	−

**Table 2:** Effect of nitrogen sources on protease production by *B. circulans *MTCC 7942.

Nitrogen supplementation (1%)^#^	Protease activity (U/mL ± SD)	Specific activity (U/mg ± SD)
Feather meal	69.5 ± 6.9	222.6 ± 21
Bovine serum albumin	216.5 ± 18.4	635.9 ± 55
Beef extract	300.6 ± 12.0	905.7 ± 41
Casein	320.7 ± 6.0	884.2 ± 114
Casamino acids	329.1 ± 13.3	966 ± 31
Yeast extract	330.1 ± 23.8	875.8 ± 163
Gelatin	339.2 ± 10.0	1063.0 ± 39
Peptone	357.0 ± 10.5	1007.0 ± 75
Soybean meal	397.0 ± 7.6	1164.0 ± 33

^#^Basal medium containing (g/L): starch, 20; KH_2_PO_4_, 1; MgSO_4_·7H_2_O, 0.05; CaCl_2_, 1; Na_2_CO_3_, 8 and cultural parameters (pH 10.0) at 37°C and 100 rpm.

**Table 3:** Effect of carbon sources on protease production by *B. circulans *MTCC 7942.

Carbon source (1%)^#^	Protease activity (U/mL ± SD)	Specific activity (U/mg ± SD)
Control	201.3 ± 10	621.6 ± 79
Xylose	223.3 ± 13	665.7 ± 58
Fructose	265.9 ± 10	940 ± 80
Sucrose	359.2 ± 7	1033 ± 32
Molasses	379.5 ± 8	1077 ± 43
Maltose	394.2 ± 4	1144 ± 22
Glucose	394.8 ± 8	1158 ± 42
Starch	406.6 ± 5	1205 ± 43

^#^Basal medium containing (g/L): soybean meal, 10; KH_2_PO_4_, 1; MgSO_4_·7H_2_O, 0.05; CaCl_2_, 1; Na_2_CO_3_, 8, and cultural parameters (pH 10.0) at 37°C and 100 rpm.
